# Propylthiouracil-Induced Acute Liver Failure: Role of Liver Transplantation

**DOI:** 10.1155/2010/910636

**Published:** 2010-12-23

**Authors:** Andres F. Carrion, Frank Czul, Leopoldo R. Arosemena, Gennaro Selvaggi, Monica T. Garcia, Akin Tekin, Andreas G. Tzakis, Paul Martin, Ravi K. Ghanta

**Affiliations:** ^1^Department of Medicine, University of Miami Leonard M. Miller School of Medicine, 185 SW 7th Street, Unit 1510, Miami, FL 33130, USA; ^2^Division of Hepatology, Department of Medicine, University of Miami Leonard M. Miller School of Medicine, Miami, FL 33130, USA; ^3^Division of Liver and Gastrointestinal Transplantation, Department of Surgery, University of Miami Leonard M. Miller School of Medicine, Miami, FL 33130, USA; ^4^Department of Pathology, University of Miami Leonard M. Miller School of Medicine, Miami, FL 33130, USA; ^5^Divisions of Hepatology and Liver and Gastrointestinal Transplantation, Department of Medicine, University of Miami Leonard M. Miller School of Medicine, Miami, FL 33130, USA; ^6^Division of Gastroenterology, Department of Medicine, University of Miami Leonard M. Miller School of Medicine, Miami, FL 33130, USA

## Abstract

Propylthiouracil- (PTU-) induced hepatotoxicity is rare but potentially lethal with a spectrum of liver injury ranging from asymptomatic elevation of transaminases to fulminant hepatic failure and death. We describe two cases of acute hepatic failure due to PTU that required liver transplantation. Differences in the clinical presentation, histological characteristics, and posttransplant management are described as well as alternative therapeutic options. Frequent monitoring for PTU-induced hepatic dysfunction is strongly advised because timely discontinuation of this drug and implementation of noninvasive therapeutic interventions may prevent progression to liver failure or even death.

## 1. Introduction

Propylthiouracil (PTU) has been implicated in drug-induced liver injury (DILI) in patients with hyperthyroidism treated with this medication. Reported injury has ranged from mild asymptomatic elevation of aminotransferases to acute liver failure (ALF). Although asymptomatic elevations in hepatic enzymes have been described in patients with untreated hyperthyroidism, recognition of hepatic dysfunction in a patient taking PTU requires immediate discontinuation of the drug and close followup.

## 2. Case Presentations

**Case 1**

**Figure 1:**
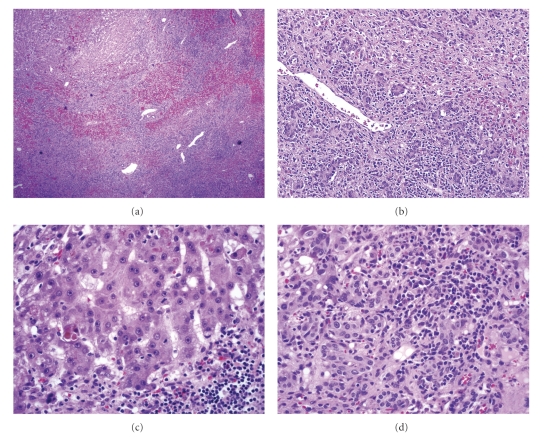
(a) Submassive confluent necrosis with hemorrhage (H & E, 40x). (b) Periportal bile duct proliferation and mixed inflammatory infiltrate (H&E, 100x). (c) Residual hepatocytes with cholestasis, acidophilic bodies, and mononuclear inflammation (H&E, 400x). (d) Eosinophilic and lymphoplasmacytic infiltrate (H&E, 400x).

A 29-year-old African American woman with Graves' disease unsuccessfully treated with methimazole was prescribed propylthiouracil (PTU) 50 mg PO every 8 hours. Aminotransferase levels were normal before therapy was started; however, mild elevations of these enzymes were initially noticed by the fourth week of therapy (AST: 64 U/L, ALT: 94 U/L, alkaline phosphatase: 170 U/L) and continued to progressively increase by week eight. By week ten of treatment, she reported jaundice, fatigue, epigastric abdominal pain, nausea, and vomiting. She denied the use of over-the-counter or herbal medications. Her past medical history and family history were not contributory. She denied excessive alcohol consumption and recreational drug use. Upon subsequent transfer to our institution, initial laboratory workup revealed a prothrombin time of 39.1 seconds, INR 3.9, total bilirubin 19.3 mg/dL, direct bilirubin 12.1 mg/dL, AST 503 U/L, ALT 443 U/L, alkaline phosphatase 509 U/L, TSH 0.013 *μ*IU/ml, and free-T4 0.7 ng/dL. Serum markers for viral hepatitis A, B, and C were negative. Antinuclear antibodies, antismooth muscle antibodies, and antimitochondrial antibodies were negative. PTU was discontinued, but the coagulopathy worsened, and she subsequently developed hepatic encephalopathy. She underwent orthotopic liver transplantation (OLT) eight days following admission. Histologic examination of the explanted liver revealed submassive, confluent necrosis with parenchymal hemorrhage, bile duct proliferation, intracellular and canalicular cholestasis, bile plugging, and severe lymphoplasmacytic and eosinophilic infiltrates. Immunostain for IgG4 was positive (Figure 1, microphotographs (a) and (b)). The patient was discharged home in stable condition on postoperative day twelve. Her liver function become normal 6 months following OLT.

**Case 2**

A 34-year-old African American woman with Graves' disease and without an antecedent history of liver disease was prescribed PTU 150 mg PO twice daily. Baseline levels of aminotransferases were normal at the time PTU was started. Six weeks later she started to complain of malaise and generalized weakness followed by progressive jaundice, but she did not seek medical care until two weeks later when she had developed confusion, nausea, and vomiting. Blood tests at that time were INR 4.3, total bilirubin 22.8 mg/dL, direct bilirubin 10.8 mg/dL, AST 1081 U/L, ALT 1227 U/L, alkaline phosphatase 272 U/L, TSH 61.2 *μ*IU/ml, and free-T4 0.6 ng/dL. Serum markers for autoimmune and viral hepatitis A, B, and C were negative. PTU was discontinued; however, her mental status continued to deteriorate with progression to severe hepatic encephalopathy. A transjugular liver biopsy revealed extensive parenchymal necrosis, collapse of the lobular architecture, bile duct proliferation, and periportal inflammation. Two days after admission, she was listed for OLT and received a liver transplant three days later. Histological examination of the native liver showed submassive confluent necrosis with prominent eosinophilic, neutrophilic, and lymphoplasmacytic infiltrate with canalicular and intracellular cholestasis and numerous lobular acidophilic bodies (Figure 1, microphotographs (c) and (d)). The allograft was retrieved from a 77-year-old deceased donor, due to the recipient's critical status. The posttransplant course was complicated by the development of graft dysfunction due to severe rejection, which did not improve with aggressive immunosuppressive therapy. The patient was relisted for OLT two weeks after the initial transplant and received a second allograft 6 days later. The postoperative course after the second transplant was complicated by a biliary leak that required reconstruction of the biliary anastomosis, as well as multiple episodes of rejection which were treated with antilymphocyte antibodies, plasmapheresis, and administration of rituximab. The patient eventually recovered. She was discharged on postoperative day 155 from the first liver transplant and is currently at home doing well 6 months after the second liver transplant.

## 3. Discussion

PTU is a thioamide derivative widely used for the treatment of hyperthyroidism which exerts its pharmacologic effects by two different mechanisms. It inhibits reactions catalyzed by the enzyme thyroid peroxidase expressed in the thyroid follicles and blocks iodine organification, and it also inhibits the enzyme 5′-deiodinase accountable for the peripheral conversion of T4 into the active T3 moiety. 

PTU-induced hepatitis was first reported by H. J. Livingston and S. F. Livingston in 1947 [1], shortly after the FDA had approved this medication for the treatment of hyperthyroidism. This patient was successfully managed with supportive care after discontinuation of the drug. Six years later, Eisen [2] reported the first case of fulminant hepatic failure attributed to PTU, an ominous adverse reaction that since then has been observed in an extremely small number of patients receiving this medication. Based on published data about the annual incidence of hypothyroidism, the reported frequency of PTU therapy (15,000 adults per year), and the incidence of PTU-induced severe liver injury (approximately 0.1% in the adult population), approximately 15 adults will develop this complication annually in USA and 10% of them (1 : 10,000 adults) will progress to ALF. Asymptomatic elevations of alanine aminotransferase (ALT) are observed in 14–28% of patients started on PTU during the first 2 months of therapy and usually resolve with no intervention [3]. Acute hepatitis associated with this medication has been reported to occur in 0.1–1.2% of patients [4]. The mortality associated with PTU-induced acute hepatitis can be as high as 25% [5]. Data suggest that the risk of severe hepatotoxicity is greater in children treated with PTU (1 : 1,000 children), but the overall incidence is significantly lower because fewer children are treated with this drug (1,500–4,000 children per year) [6]. For example, a significant reduction in PTU use by pediatric endocrinologists has been observed over the past several years, and some authors advocate that PTU should never be used as a first-line therapy in children due to the potential risk severe hepatotoxicity [7]. 

Liver biopsy remains the gold standard for diagnosis of PTU-induced hepatic injury, but the diagnosis is often inferred from the time course after initiation of PTU therapy [5, 8]. PTU hepatotoxicity has been reported to cause a variety of histological changes including portal and periportal inflammation with eosinophilic, lymphocytic, and plasmacytic infiltration in varying combinations, chronic active hepatitis, and submassive or massive hepatic necrosis [9]. These histological features resemble autoimmune hepatitis type 1 (AIH-1) and are referred to as drug-induced AIH-1. A multifactorial hypothesis has been proposed as an explanation for PTU-induced hepatopathy in which inhibition of glucuronyl transferase, reduced bile acid synthesis, and increased oxygen consumption by the hepatocytes could be implicated in more or less extent [10, 11]. Severe PTU-induced hepatotoxicity is postulated to be a dose independent, idiosyncratic hypersensitivity reaction. This theory is supported by positive lymphocyte sensitization studies [3, 4, 12]. Some authors have noted an association between this pathologic condition and positive antineutrophil cytoplasmic antibodies (ANCA); however, ANCA positivity has been reported in up to 50% of asymptomatic patients receiving PTU, and this finding has been considered to be incidental in the majority of cases and not directly related to DILI or ANCA-associated vasculitis [13, 14]. 

Treatment options for PTU-induced liver failure are limited. Immediate discontinuation of the drug is imperative, but progression of liver injury may occur, particularly in cases with severe forms of DILI, and aggressive supportive care is usually required. The milder form of PTU-induced hepatotoxicity, characterized by symptomatic hepatitis, has been associated with complete recovery after discontinuation of the drug [15]. This outcome has also been reported in cases with cholestatic liver injury, although the time required for normalization of biochemical markers was longer in the latter group [4]. Some authors have suggested that the coincidental clinical improvement in patients receiving systemic corticosteroids for the treatment of thyrotoxicosis might reflect a therapeutic response [16]. Plasmapheresis was apparently successfully used, in one case report of fulminant hepatic failure; nonetheless, concomitant use of prednisone confounded evaluation [8]. Liver transplantation has been reported in severe, life-threatening cases of PTU-induced hepatotoxicity with good outcomes [5, 15]. Between 1990 and 2007, the United Network for Organ Sharing (UNOS) reported 23 liver transplants (16 in adults and 7 in children) for PTU-induced ALF. Population-based estimates of liver transplantations for ALF using the UNOS database indicate that drug-induced ALF accounts for approximately 12–15% of liver transplants for ALF in USA [17, 18]. PTU is the third most common single drug responsible for DILI requiring liver transplantation in the general population, preceded by acetaminophen and isoniazid in the adult population and by acetaminophen and valproic acid in pediatric patients [17]. The mean age of patients with PTU-induced ALF is 28 years; over two thirds of these patients are females and almost half of them are African Americans. Therefore, African American ethnicity may be a risk factor for severe form of PTU-induced hepatotoxicity; nonetheless, there are no data supporting ethnicity-specific or genetic variations responsible for these observations, and specific recommendations about the use of PTU in African Americans cannot be made. ALF secondary to PTU and herbal substances is associated with higher bilirubin levels (23.2 mg/dL and 23.6 mg/dL on average, resp.) compared to other drugs, including acetaminophen (9.8 mg/dL on average). The one-year graft survival rate after liver transplantation for PTU-induced ALF is 84%; however, no significant difference has been found in graft survival rates after ALF caused by PTU, acetaminophen, isoniazid, or phenytoin [17]. 

We add to the body of literature two more cases of severe PTU-induced liver failure successfully treated with OLT. The first patient had an uneventful posttransplant recovery, but the second case had a complicated clinical course requiring retransplantation and multiple interventions to prevent loss of the second allograft. Extended criteria for donor organs, including advanced-age donors, may be used due to the emergency need for OLT in patients with fulminant hepatic failure; nonetheless, higher rates of severe complications requiring specialized care underscore the importance of treating these patients in experienced, high-volume transplant centers. There are no official recommendations regarding monitoring for serologic markers of liver injury during PTU therapy; however, some authors have recommended monthly monitoring of serum transaminases for the first six months of therapy, which is the period where most cases of PTU-induced DILI occur [19, 20]. Although there are no evidence-based data to support this recommendation, we agree with the suggested time intervals and duration of monitoring and emphasize the importance of early discontinuation of therapy when early signs of liver injury are detected.

## 4. Conclusions

Clinicians taking care of patients being treated with PTU should have a low threshold for suspecting medication-related adverse reactions, particularly for PTU-induced DILI. Routine monitoring of serum transaminases and close followup is recommended; nonetheless, the appropriate intervals and duration of followup have not been established. Early identification of clinical signs and abnormalities in biochemical markers of hepatic injury followed by immediate discontinuation of PTU, aggressive supportive care, and transfer of patients to centers capable of performing emergent liver transplantation is strongly advised due to the high mortality associated with severe forms of this complication.
